# Case Report: Successful treatment of refractory generalized pustular psoriasis with xeligekimab: a report of two cases

**DOI:** 10.3389/fimmu.2026.1748050

**Published:** 2026-06-12

**Authors:** Xiao Zang, Xiaoxuan Shi, Huijie Li, Na Wang, Qing Yang

**Affiliations:** 1 Department of Dermatology, Shandong Provincial Hospital of Dermatology, Jinan, China; 2 Shandong Provincial Institute of Dermatology and Venereology, Shandong Academy of Medical Sciences, Jinan, China

**Keywords:** generalized pustular psoriasis, IL-17A inhibitor, psoriasis, treatment, xeligekimab

## Abstract

Generalized pustular psoriasis (GPP) is a severe, relapsing skin disorder characterized by widespread erythema and sterile pustules. Classical systemic therapies often yield suboptimal results. Xeligekimab is a novel, fully human anti-interleukin-17A (anti-IL-17A) monoclonal antibody recently approved in China for plaque psoriasis. Its efficacy in GPP has not been previously reported. This report presents two cases of refractory GPP successfully treated with xeligekimab. Both patients- a 25-year-old male and a 70-year-old male had long-standing GPP that was unresponsive to conventional systemic therapies including acitretin and methotrexate. After receiving a single 200 mg dose of xeligekimab, both patients exhibited rapid and significant clinical improvement within 3 to 4 days. Remarkably, one patient remained in remission at the 3-month follow-up after only one injection. This is the first report to suggest that xeligekimab is a potent and promising therapeutic option for inducing rapid and sustained remission in patients with GPP.

## Introduction

Generalized pustular psoriasis (GPP) is a severe subtype of psoriasis, its typical clinical features include the acute onset of erythematous skin with coalescing sterile pustules, often accompanied by systemic symptoms such as fever, chills, cutaneous pain, leukocytosis, and elevated C−reactive protein (CRP) and erythrocyte sedimentation rate (ESR). Recurrent flares are associated with progressive organ impairment and potential life-threatening complications. Traditional first-line systemic therapies, including acitretin, cyclosporine A (CsA), or methotrexate (MTX), have notable limitations: a slow onset of action, modest efficacy, and considerable adverse drug reactions (1). In recent years, the development of biologic agents has revolutionized the therapeutic landscape for GPP (2).

IL-17A is a pivotal pro-inflammatory cytokine highly overexpressed in GPP lesions, playing a central role in neutrophil recruitment, pustule formation, and amplification of the inflammatory cascade. Emerging evidence supports the efficacy of IL-17 pathway inhibitors, including secukinumab and ixekizumab, in the management of GPP, although high-quality evidence remains limited. Spesolimab is the first and only biologic agent approved specifically for GPP, targeting the interleukin 36 receptor (IL-36R), a master regulator of GPP pathogenesis. Its approval was based on the pivotal Phase II Effisayil 1 trial, which demonstrated significant pustule clearance; however, high cost and logistical challenges restrict its real−world accessibility.

Xeligekimab is a novel fully human anti-IL-17A monoclonal antibody recently approved in China for moderate-to-severe plaque psoriasis. Its efficacy and safety profile in GPP have not been reported. The primary objective of this case series is to describe the clinical outcomes of xeligekimab in two patients with refractory GPP, providing real-world evidence for this emerging therapeutic strategy.

## Case reports

### Case 1

A 25-year-old male presented with a 2-week flare of generalized erythema and pustules, following an 11-year history of the condition. The initial onset occurred at age 14, manifesting as diffuse cutaneous erythema and pustulation that resolved with oral acitretin capsules 30mg daily. Although the lesions resolved initially, the disease followed a relapsing-remitting course; however, the patient achieved nearly a decade of sustained remission with maintenance therapy. The patient had no significant comorbidities, medication/food allergies, or family history of psoriasis.

**Figure 1:**
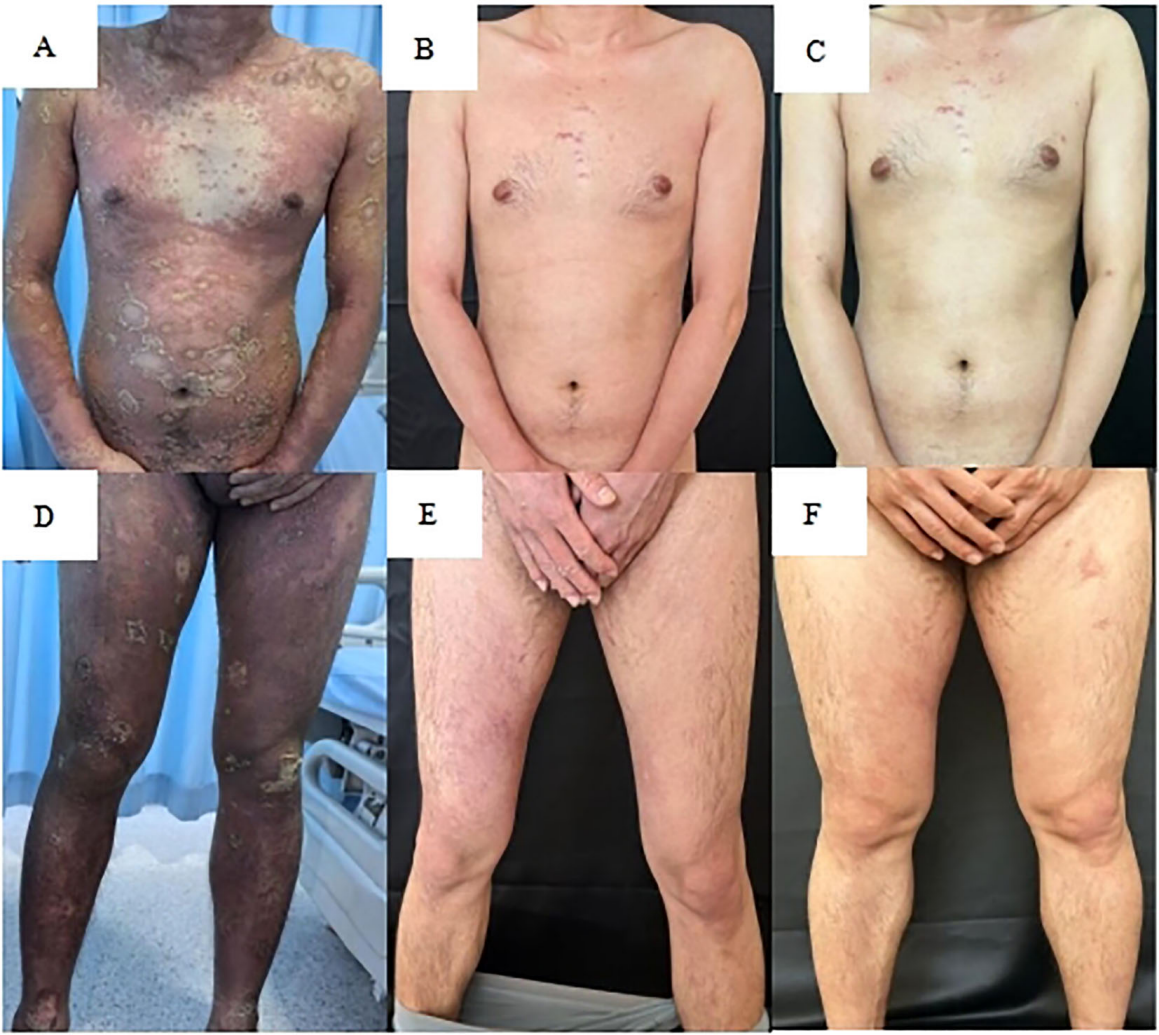
CASE 1 **(A, D)** widespread cutaneous erythema and coalescing pustules on day 0; **(B, E)** pustules resolved completely with residual erythema at week 2; **(C, F)** erythema resolved completely.

Five days before admission to our hospital, the patient reported worsening symptoms, including erythema and an increase in disseminated body pustules, which showed a suboptimal response to oral acitretin 30 mg daily. He described feeling increasingly anxious and frustrated, as the treatment that had kept him stable for years no longer seemed to work. He noted that the pustules were not only visually distressing but also caused significant itching and tenderness, interfering with his sleep and daily activities. After 9 days of therapy, erythema and pustules became generalized across the whole body (**Figures 1A, D**), accompanied by fever, chills and cutaneous pain. The patient stated that he felt “completely overwhelmed” by the rapid worsening, and he decided to seek emergency care at our hospital because he could no longer tolerate the pain and feared the disease might become uncontrollable. Laboratory results revealed leukocytosis (white blood cell count [WBC]: 17.86×10^9^/L), with normal C-reactive protein (CRP), erythrocyte sedimentation rate (ESR), and tuberculosis interferon-gamma release assay (TB-IGRA). Detailed laboratory data are summarized in **Table 1**.

**Table 1:** Laboratory parameters before and after xeligekimab treatment.

Parameter	Case 1 (baseline)	Case 1 (post-treatment day 4)	Case 2 (baseline)	Case 2 (post-treatment day 3)	Normal reference range
WBC (×10^9^/L)	17.86	6.82	16.55	7.15	3.5–9.5
CRP (mg/L)	1.7	0.65	154	12.3	0–10
ESR (mm/h)	6	5	42	15	0–15
Hemoglobin (g/L)	149	155	113	125	120–160
Albumin (g/L)	48.4	45.2	26.6	35.8	35–55
BSA	90%	20%	30%	15%	1-100%
GPPGA	3	1	3	1	1-4
GPPASI	37.8	7.2	15.8	4.8	0-72

WBC, white blood cell; CRP, C-reactive protein; ESR, erythrocyte sedimentation rate.

After obtaining written informed consent, treatment was switched to subcutaneous xeligekimab 200 mg. Four days after xeligekimab administration, the erythema and pustules had almost completely resolved. The patient reported feeling “a dramatic sense of relief” as the skin pain and fever subsided rapidly. He remarked that he could see visible improvement within 48 hours, which greatly boosted his confidence in the treatment. No treatment emergent adverse events, including injection-site reactions, infection, or hepatorenal dysfunction were observed. The patient continued xeligekimab 200mg every two weeks as maintenance therapy and remained flare-free through the 3-month follow-up (**Figures 1B, C, E, F**). During follow-up visits, he expressed gratitude for regaining his quality of life, noting that he could return to work and social activities without the constant fear of a flare. He also mentioned that the rapid response to xeligekimab exceeded his expectations. Notably, prior acitretin exposure may represent a potential confounding factor for the observed therapeutic response.

### Case 2

**Figure 2:**
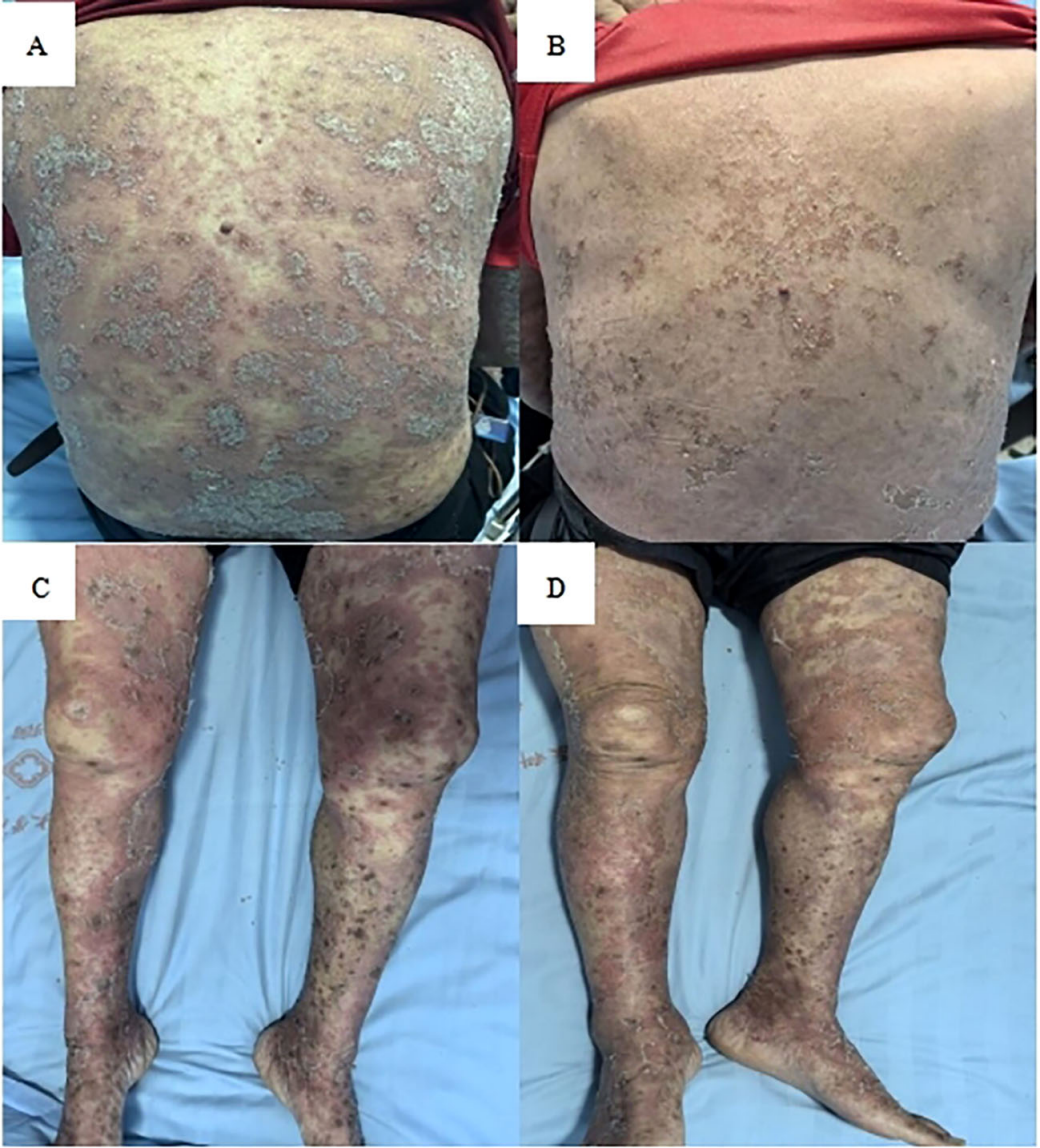
CASE 1 **(A, C)** widespread cutaneous erythema and coalescing pustules on day 0; **(B, D)** pustules resolved completely with residual erythema and exfoliation at day 3.

A 70-year-old man with a 10-year history of plaque psoriasis developed recurrent generalized pustulation over the preceding five years. Previous treatments included methotrexate 15 mg weekly and acitretin 30 mg daily, which achieved partial pustule resolution but were associated with rapid relapse upon discontinuation. CsA was contraindicated due to a 20-year history of hypertension. The patient reported that the recurrent pustulation episodes had gradually eroded his quality of life; he felt “trapped” by the need for ongoing medication and frustrated by the quick return of symptoms whenever treatment stopped. One month prior to presentation, diffuse edematous erythema and generalized pustules recurred, with partial desquamation of older plaque lesions. The patient received oral acitretin 30 mg daily for 20 days, but the response was suboptimal, with persistent new erythematous lesions and pustules formation (**Figures 2A, C**). Laboratory studies revealed leukocytosis (WBC 16.55×109 cells/L), mild anemia (low hemoglobin, 113g/L), hypoalbuminemia (26.6 g/L), elevated ESR (42 mm/h) and markedly elevated CRP (154 mg/L). Detailed laboratory data are summarized in **Table 1**.

Therapy with xeligekimab (200 mg, subcutaneous injection) was initiated after obtaining the patient’s informed consent, and acitretin was discontinued. Dramatic clinical improvement was observed within just three days, characterized by fading of edematous erythema on the trunk and extremities and complete resolution of pustules, followed by desquamation. No clinically significant adverse events were detected during treatment. The patient was discharged in improved condition. Although maintenance xeligekimab was not administered after discharge, complete resolution of skin lesions without relapse was documented at the 3-month follow-up. During the follow-up visit, the patient shared that the three months without any skin lesions were “the best months in the past five years.” He was able to resume daily activities, including gardening and spending time with grandchildren, without embarrassment or pain. He expressed willingness to receive xeligekimab again if a future flare occurs.

## Discussion

GPP is a severe, recurrent, and debilitating inflammatory disorder associated with substantial morbidity and potential life-threatening complications. Current conventional systemic therapies including acitretin, Mtx, and CsA, have limited efficacy, delayed onset of action, and unfavorable safety profiles, highlighting an unmet need for more effective and better-tolerated therapies. In recent years, numerous biologic agents, including tumor necrosis factor-alpha (TNF-α) inhibitors (3) (including infliximab (4), adalimumab (5), etc.) and IL-17 inhibitors (6) (including secukinumab, ixekizumab (7), etc.)have been reported effective for GPP, although most evidence is derived from case reports or small case series.

Spesolimab is currently the only biologic approved for GPP treatment, acting via selective blockade of the IL-36R, a pathway central to GPP pathogenesis. Its approval was supported by the pivotal Phase II Effisayil 1 trial, in which 54% of patients achieved complete pustule clearance following a single dose, establishing a new therapeutic benchmark. Nonetheless, the high cost and the practical administration difficulties resulting from the intravenous route of administration limit its widespread clinical use.

Both patients had a long disease history, and previous treatments, including acitretin and/or methotrexate, were either ineffective or provided only temporary relief, with rapid recurrence upon discontinuation, greatly affecting their quality of life. During this hospitalization, biologic agents were considered; however, both patients declined spesolimab due to its prohibitive cost.

IL-17A is a key driver of psoriatic inflammation, promoting neutrophil chemotaxis, pustule formation, and release of pro-inflammatory mediators, with marked upregulation in GPP lesions. IL−17A inhibitors selectively neutralize IL−17A, rapidly suppressing inflammation and resolving cutaneous lesions (8) Xeligekimab (Jinlixi^®^) is a recombinant fully human interleukin (IL)-17A-neutralizing immunoglobulin G4 (IgG4) monoclonal antibody approved in China on 27 August 2024 (9) for the treatment of adult patients with moderate-to-severe plaque psoriasis who are candidates for systemic therapy or phototherapy. As a relatively novel IL-17 inhibitor, its efficacy in pustular psoriasis has not been reported.

Previous case reports and small series indicate that secukinumab and ixekizumab induce rapid improvement in GPP, consistent with observations in the present study. As a fully human IgG4 antibody, xeligekimab is expected to have low immunogenicity and favorable safety potential relative to other IL-17 inhibitors.

This study has several strengths. To our knowledge, it is the first report to demonstrate the efficacy of xeligekimab in refractory GPP, thereby filling an important gap in the literature. Both patients achieved marked clinical improvement within 3-4 days after a single 200 mg dose, which is substantially faster than conventional systemic therapies (typically weeks to months) and comparable to the rapid onset reported for other IL-17 inhibitors. Notably, one patient (Case 2) remained in complete remission for three months without any maintenance therapy, suggesting a durable effect that merits further investigation. No treatment-emergent adverse events were observed, supporting the favorable short-term safety profile of xeligekimab. The inclusion of patient perspectives adds valuable insight into the real-world impact of treatment on quality of life and treatment satisfaction.

This study has several limitations: The sample size is limited to two cases, and the 3 months follow-up is insufficient to assess long-term efficacy and safety. Prior acitretin exposure in Case 1 may confound the attribution of clinical improvement to xeligekimab, but the acitretin pharmacokinetic characteristics suggest that residual drug effects are unlikely to significantly affect the rapid response observed after xeligekimab treatment. The diagnosis of GPP has some limitations, including the lack of skin biopsy for histopathological confirmation, the absence of genetic testing for IL36RN mutations, and the reliance on clinical diagnosis based on typical GPP features.

In summary, this is the first report describing the successful use of xeligekimab in refractory GPP. Xeligekimab induced rapid and sustained clinical remission in patients with GPP refractory to conventional therapies and ineligible or unwilling to receive approved biologic therapy, representing a promising alternative for real-world clinical practice. However, given the small sample and limited follow-up, these findings are preliminary and require confirmation in large-scale, prospective, randomized controlled trials to establish efficacy, safety, and long-term outcomes.

## Data Availability

The original contributions presented in the study are included in the article/supplementary material. Further inquiries can be directed to the corresponding authors.
